# Butterfly Conservation in China: From Science to Action

**DOI:** 10.3390/insects11100661

**Published:** 2020-09-25

**Authors:** Wen-Ling Wang, Daniel O. Suman, Hui-Hong Zhang, Zhen-Bang Xu, Fang-Zhou Ma, Shao-Ji Hu

**Affiliations:** 1 Yunnan Key Laboratory of International Rivers and Transboundary Eco-Security, Yunnan University, Kunming 650500, China; wangwl@ynu.edu.cn; 2 Institute of International Rivers and Eco-Security, Yunnan University, Kunming 650500, China; 3 Rosenstiel School of Marine and Atmospheric Science, University of Miami, Miami, FL 33149, USA; dsuman@rsmas.miami.edu; 4 School of Agriculture, Yunnan University, Kunming 650500, China; leozhanghh@foxmail.com (H.-H.Z.); zhenbangxu@mail.ynu.edu.cn (Z.-B.X.); 5 Nanjing Institute of Environmental Sciences, Ministry of Ecology and Environment of the P.R.C., Nanjing 210042, China; mfz@nies.org

**Keywords:** Lepidoptera, pollinator, protected species, refugia, agroecosystems, urban greening, butterfly ranching and farming, citizen science, public awareness

## Abstract

**Simple Summary:**

Butterflies provide numerous ecological and socio-economic services and are important indicator species. China is home to over 2000 species of butterflies and in recent years has elevated biodiversity conservation on the national agenda. This manuscript reviews China’s butterfly conservation efforts and its legal and policy frameworks. We note some of the current limitations in butterfly conservation (inappropriate listing of protected species; over-reliance on inventories, rather than holistic research) and offer numerous recommendations to improve conservation efforts. Our recommendations include those related to integration of scientific data into policy (designation of scientifically-based protected areas; development of appropriate criteria for classifying protected species; use of umbrella species for conservation purposes), adoption of butterfly-friendly land use policies in rural and urban areas, butterfly ranching and farming, use of citizen science to improve data collection, and enhanced public outreach and environmental education campaigns. Our recommendations will help further the goals of China’s National Biodiversity Conservation Strategy and Action Plan (2011–2030).

**Abstract:**

About 10% of the Earth’s butterfly species inhabit the highly diverse ecosystems of China. Important for the ecological, economic, and cultural services they provide, many butterfly species experience threats from land use shifts and climate change. China has recently adopted policies to protect the nation’s biodiversity resources. This essay examines the current management of butterflies in China and suggests various easily implementable actions that could improve these conservation efforts. Our recommendations are based on the observations of a transdisciplinary group of entomologists and environmental policy specialists. Our analysis draws on other successful examples around the world that China may wish to consider. China needs to modify its scientific methodologies behind butterfly conservation management: revising the criteria for listing protected species, focusing on umbrella species for broader protection, identifying high priority areas and refugia for conservation, among others. Rural and urban land uses that provide heterogeneous habitats, as well as butterfly host and nectar plants, must be promoted. Butterfly ranching and farming may also provide opportunities for sustainable community development. Many possibilities exist for incorporating observations of citizen scientists into butterfly data collection at broad spatial and temporal scales. Our recommendations further the ten Priority Areas of China’s National Biodiversity Conservation Strategy and Action Plan (2011–2030).

## 1. Introduction

Butterflies constitute a conspicuous group of arthropods with around 19,000 species widespread throughout all Earth’s fauna [1,2]. Besides being a beautiful component of biodiversity and an indicator of environmental health and change [3,4,5,6], butterflies provide numerous ecosystem services, as well as direct benefits to humans, especially through the following roles: (1) pollinators to over 20,000 species of wild and domesticated plants, second only to bees and wasps [7,8], (2) providers of natural products (pigments and metabolic products) which could be candidates for new dyes and medicines [9,10], (3) bionic models for industrial research and development, including hydrophobic materials, aerodynamic structures, and photo-electronic materials [11,12], (4) model organisms for environmental and biological research (e.g., mimicry, development, genetics, evolution, population dynamics, and conservation) [13,14,15,16,17,18,19,20,21,22,23,24,25,26,27,28,29], (5) design patterns and aesthetic uses in different cultures for centuries, including garden and landscape planning [30,31,32,33,34,35], and (6) butterfly eco-tourism that attracts numerous tourists around the world each year, bringing significant income to local citizens [36].

China is a great mega-biodiversity hotspot in Asia [37,38], where butterflies have long been utilised as a natural resource in various ways. Yet, China is also a region heavily impacted by population and environmental stresses [39,40], posing cumulative threats to butterfly diversity. Recently with the implementation of China’s policy of “ecological civilisation” [41,42], biodiversity conservation has been brought to the attention of the nation’s planners as a component to attain the goal of sustainable development [43,44,45]. Conservation of butterflies has been listed as one of the priorities for arthropod resources by the Ministry of Ecology and Environment (formerly the Ministry of Environmental Protection before 2018), and a nation-wide butterfly monitoring network (China BON-Butterflies) ) (BON: Biodiversity Observation Network) was established in 2016 [46].

Compared to some European, North American, and Asian countries, which count over 40 years of butterfly conservation efforts, China’s butterfly conservation initiatives are still in their initial stages [47]. Although the “*Law of the People’s Republic of China on the Protection of Wildlife*” (hereafter, *Wildlife Protection Law*) [48] provides an initial legal framework, China still lacks a coherent policy for butterfly conservation. The absence of such policy not only makes conservation measures difficult to implement under varied scenarios, but also creates a significant gap between the framework legislation and practical cases related to non-protected butterfly species. Therefore, formulation of practical butterfly conservation policies is the first critical step to enhance future butterfly protection in China.

This article first provides an overview of butterfly diversity and its major threats in China. We then examine the current conservation measures in China and identify gaps in research and applications, as well as the challenges and barriers to integration of research into conservation policy formulation. Based on our analyses and evaluation, we recommend measures to bridge the gaps and overcome the obstacles and barriers to move the conservation measures ahead drawing on examples from around the world. Our findings help improve understanding of the challenges faced by butterfly conservation in China, suggest the scientific framework for future conservation-related research, and additionally provide a roadmap for the formulation of butterfly conservation policies.

## 2. China’s Butterfly Diversity and Threats

China has an extraordinary butterfly diversity among East Asian countries, with 2077 species recorded to date [49]. Apart from high species richness, China’s butterfly diversity is also characterised by high regional endemism. Our analysis identified 779 endemic or narrow-ranged species (37.5%) confined in West and Southwest China [49].

Most butterflies are oligophagous insects which only associate with a limited number of closely related larval food plants, thus making them generally very sensitive to environmental disturbance [2,50,51,52]. This phenomenon is more typical in regional endemic and narrow-ranged species than in widespread ones (cosmopolitans), as the latter constantly evolve better adaptation strategies to utilize different larval food plants across their distribution range [53].

Globally, habitat loss is the most imminent threat to butterfly diversity; deforestation (including agriculture development), urban expansion, and infrastructure construction are thought to be three major factors [2,54,55,56,57,58]. In China during the past five decades, habitat loss associated with deforestation has posed a constant threat to biodiversity particularly before 1998 when the national forest conservation project was initiated significantly reducing deforestation [40,59,60]. Nevertheless, urban expansion and infrastructure construction continue to be important drivers of butterfly habitat losses. Harvest of certain wild plants for traditional Chinese medicine may also impact habitat quality in some areas, especially in regions where narrow-ranged butterflies are very dependent on targeted plants [61]. For instance, several studies suggest that the population decline of *Bhutanitis* spp. in West China is associated with the prolonged harvest of *Aristolochia* spp. (pipevines) [62,63,64].

Plant protection products (PPPs, e.g., pesticides and herbicides) associated with agriculture pose a second threat. Apart from imminent mortal effect after acute exposure, the larval performance, species richness, and abundance are also affected by chronical exposure [65,66,67,68,69,70]. Therefore, butterflies often suffer collateral damage under broad-spectrum pesticides in either agroecosystems or artificial green space in urban areas [2,71,72,73]. Although China has begun to reduce the usage of PPPs since 2017 [74,75], this threat will still continue in the near future.

Commercial butterfly collection is the third potential threat, especially in western montane and southern tropical areas inhabited by endemic (sought-after by collectors) and ornamental (used for ceremonial release or decoration) species. Despite butterflies being R-strategy organisms, their population abundance would likely decline were prolonged mass hunting in a specific area left unregulated, especially for those narrow-ranged, regionally endemic, and univoltine species (one generation per year) or, additionally, when the above-mentioned threats combine with commercial collection in a same area [2].

An additional driver that may threaten some butterfly species is global climate change. Initial research also suggests that global warming is responsible for shifts in lower-latitude butterfly species to higher latitudes [76,77]. Endemic species inhabiting narrow ranges, particularly in mountainous regions of Southwest China and the Himalayas, may be particularly vulnerable to climate change impacts as they may have limited area for migration to higher elevations [78].

## 3. China Butterfly Conservation: Current Situation and Gaps

### 3.1. Current Conservation Modes

#### 3.1.1. Protected Areas

Protected areas in China include both national and provincial nature reserves, as well as forest parks and national scenic areas. These areas were established and managed by forestry and environment protection authorities mainly for protection of certain endangered species, such as rare plants and higher animals (charismatic vertebrates) (e.g., giant panda, crested ibis, green peacock, etc.), or for protection of representative ecosystems, biota, and geological structures [79]. Over 17% of China’s land area enjoys some designation as protected area [80].

Although butterflies are not the primary focus in most protected areas, butterfly diversity is greater inside protected areas because these areas have sustained much less human disturbance (e.g., deforestation, agriculture-related activities including PPP application, and infrastructure or construction projects). Moreover, official permits must be obtained in advance of any necessary activities inside these areas, and commercial collection is strictly prohibited [81]. Recent butterfly monitoring revealed higher species richness and population abundance of various butterfly fauna inside protected areas compared to neighbouring unprotected areas [82] (unpublished work).

Several protected areas have been established with the express goal of protecting butterflies, even though some of these are contiguous with or include portions of existing conservation areas. The first protected area for butterflies was established in 1989 in Niushoushan near Nanjing to protect the population, habitat, and host plant of *Luehdorfia chinensis* (Z.H. Li, pers. comm.). In recent years, the Meihuashan (Guangdong Province) and Wuyanling (Zhejiang Province) Nature Reserves were established for the iconic *Teinopalpus aureus* in China. Similarly, a conservation area for *Bhutanitis* spp. inside the Yulong Xueshan Nature Reserve in Yunnan Province was also established in 2018.

Another important contribution of protected areas to butterfly conservation is that many protected areas regularly conduct faunistic inventories/surveys, producing a good number of butterfly inventories that benefit scientific research and conservation practices. The recent faunistic survey in Gaoligongshan National Nature Reserve even rediscovered several unique and rarely seen taxa further increasing the butterfly species richness of this nature reserve [83].

#### 3.1.2. Legislation and Protected Species Lists

China acceded to the Convention on International Trade of Endangered Species of Wild Flora and Fauna (CITES) on 1 August 1981 and has been an active member since then. CITES Appendix I lists 5 species of Papilionidae (swallowtails/birdwings), none which are present in China. However, some 41 species of Papilionidae have been listed in Appendix II, and 9 of these are present in China (*Parnassius apollo*, *Bhutanitis thaidina*, *B. lidderdalii*, *B. manfieldi*, *B. ludlowi*, *Teinopalpus aureus*, *T. imperialis*, *Troides aeacus*, and *T. helena*)—particularly in tropical southern regions and Himalayan areas of the country [84,85].

The *Wildlife Protection Law* and its regulations provide the second measure for wildlife protection and management. The goals of this legislation are to protect, develop, and rationally utilize species that are rare and face extinction (Art. 1) through direct protection, active breeding, and scientific research (Art. 4). Responsibility to administer wildlife resources exists at all levels of government—Central, Provincial, County, and Municipal (Arts. 6 & 7). The State Council created two lists: Class I species are near extinction, while Class II species are rare (Art. 8). Capturing listed wildlife is prohibited (Art. 8)—except by permit for purposes of scientific research, exhibition, or breeding from either the Central Government administration (Class I) or the Provincial or Municipal Government (Class II) (Art. 16). Collection of listed species in natural reserves is expressly prohibited (Art. 18). The legislation also establishes enforcement mechanisms for violations that may lead to criminal prosecution, confiscation, and/or fines, as well as injunctions (Arts. 32 & 34). In practice, the *Wildlife Protection Law* generally stipulates that construction projects must avoid protected areas (Art. 20) and that construction projects causing adverse environmental impacts to wildlife under special protection must undergo environmental impact assessment review (Art. 12). Conservation measures, such as wildlife corridors and habitat restoration, must be designed when a project cannot completely avoid adverse environmental impact to listed species. However, we know of no example of project mitigation because of the existence of a listed butterfly species, especially when these butterflies inhabit habitats outside protected areas.

In 1989, China issued the “*List of Wildlife under Special State Protection*” (hereafter, “*SSP List*”) categorising protected species as either Class I or II. The *SSP List* contains only five Papilionidae species of butterflies (0.24% of total known species in China), with *Teinopalpus aureus* being Class I and four other species, namely *Bhutanitis thaidina*, *B. mansfieldi*, *Luehdorfia chinensis*, and *Parnassius apollo*, listed as Class II [86]; (SSP List, 2006). Good correlation exists between the *SSP List* and the CITES Appendices; *T. aureus*, *B. mansfieldi*, *B. thaidina*, and *P. apollo* all appear in Appendix II.

In 2000, China promulgated the “*List of Terrestrial Wildlife under State Protection for Ecological, Economic and Scientific Values*” (hereafter, the “*EES List*”) as a supplement to the *SSP List*, containing 89 additional butterfly species of all families (4.2% of total known species in China) [87]. The two lists, in association with the *Wildlife Protection Law*, not only clarify the protection level of listed species and relevant penalties for violators, but also provide standards for judicial interpretation and law enforcement. They are undoubtedly legislative benchmarks for China’s butterfly conservation.

Direct capture of listed butterfly species entails significant differences between the two lists. Collection of both live and dead specimens of species listed on the *SSP List* requires permits issued by the appropriate forestry administration. Specifically, the wildlife protection authority of the State Council grants the permits for Class I species, while the provincial Forestry and Grassland Administrations issue permits for Class II species [48]. However, species appearing on the *EES List* only require permits issued from County level authorities [48].

#### 3.1.3. National Biodiversity Conservation Strategy

In September 2010 the Ministry of Environmental Protection and the State Council approved the China National Biodiversity Conservation Strategy and Action Plan (2011–2030) (hereafter, “China Biodiversity Action Plan”) to implement the provisions of the Convention on Biological Diversity, improve conservation efforts, and confront emerging challenges to the nation’s biodiversity [80]. The cross-sectoral document lists a number of strategic tasks that are highly relevant to butterfly conservation: conducting baseline surveys and inventories and raising public awareness about biodiversity conservation and establishing a system of public monitoring. Perhaps the Plan’s most important Priority Action Areas for butterfly conservation are carrying out identification, monitoring, and evaluation of biodiversity (Priority Area 3), strengthening in-situ biodiversity conservation inside and outside nature reserves (Priority Area 4), coping with climate change and assessing impacts on key ecosystems and species (Priority Area 8), and establishing public participation mechanisms for biodiversity conservation (Priority Area 10). However, butterfly conservation is relevant to all ten Priority Areas.

To implement the China Biodiversity Action Plan for Lepidoptera, the MEP initiated the China Butterfly Diversity Observation Network (China BON-Butterflies) in 2016 [46] to determine a baseline for spatial distribution of butterflies in China. At its initiation, the project collected observations in 117 standardized sample regions (629 standardized transects) over 31 provinces, autonomous regions, and municipalities.

### 3.2. Gaps and Challenges

#### 3.2.1. Lack of Holistic Conservation Research

Although a large volume of butterfly inventories reported in Chinese protected areas exists, conservation research involving analyses of human-butterfly interactions, user conflicts, impacts of climate change and land use changes on butterfly distributions, or population refugia is still very limited. Conservation analyses have tended to focus on some iconic protected butterflies species, such as *Teinopalpus aureus*, *B. thaidina*, and *Troides aeacus* [62,78,88,89]. Research efforts rarely examine other non-protected butterflies in a broader sense. The lack of such research has already produced a bottleneck for butterfly conservation efforts due to the limited data available to conservationists and policy makers.

#### 3.2.2. Bias in Protected Species

The second gap is the lack of correlation between the protected status of butterflies and their actual abundance and endemism. For instance, the National Class I *T. aureus* occupies much a wider distribution range than the National Class II *B. thaidina* and *B. mansfieldi*, as well as being non-endemic [49,90,91]. Furthermore, this flagship species-based mechanism has resulted in the failure to grant protected status to many narrow-ranged, locally endemic species, including a number of Theclini hairstreaks (Lycaenidae), confined to the montane regions of Southwest China and strongly associated with the broadleaf forest biota [51,86,87].

#### 3.2.3. Unregulated Commercial Collection

Another challenge is commercial collection, including its downstream business chain. As mentioned above, commercial collection of some ornamental and endemic species can pose threats to these taxa, either in ‘low value/high volume’ or ‘high value/low volume’ modes [2]. Local residents in some remote rural areas consider that butterfly collecting provides an important financial compensation to their household—typically in tropical southern Yunnan, where butterfly collecting has occurred for over two decades and become part of the ‘routine labour’ of local residents (L. Ai & L. Bo, pers. comm.)

Similar situations were reported in many other regions globally (e.g., Central Asia, Nepal, Far East Russia, Indonesia, the Philippines, Papua New Guinea, tropical America and tropical Africa) [92,93,94]. Unlike butterfly smuggling of protected species, which can be effectively intercepted by law enforcement agencies [95], commercial collection and the downstream business chain are more difficult to regulate. The very limited numbers of enforcement actions against butterfly businesses in recent decades have all involved protected butterfly species [95,96]. Although China’s wildlife authorities discourage such collecting [48,97], professional butterfly dealers in the business chain can constantly make sufficient profit based on the butterflies collected by locals, as long as species found in the *SSP List* or the *EES List* are not involved.

## 4. Recommendations of Future Butterfly Conservation

### 4.1. Improving Conservation Science

#### 4.1.1. Revising the Protected Species Lists

According to the *Wildlife Protection Law*, China should evaluate and revise the *SSP List* every five years (Art. 11) [48], thus providing an opportunity for revision and adaptation of the protected butterfly list with the advance of scientific studies. Adding and removing species, as well as rank adjustment are equally important for effective conservation outcomes. The listing of species as Class I or II must take species’ survival status and future changes into consideration. Survival status includes population size, genetic diversity, and distribution range; while future changes are largely correlated to human disturbance and climate change.

For butterflies, distribution ranges can represent their survival status better than population sizes, which are difficult to assess. Narrow-ranged species are logically more vulnerable to any disturbance than widespread ones; therefore, the first step of species selection should focus on the narrow-ranged species in all families (not only restricted to the iconic Papilionidae). A hierarchical ranking system of Natural Heritage previously used by tiger beetles in Yunnan could be adopted to facilitate this step [98,99]. Human disturbance can be measured by past or continuing events using geographic information system (GIS) technologies (see Section 4.1.2.). A species’ response to climate change can also be effectively evaluated using GIS-based models with future climate projections [78,100,101,102,103,104,105,106]. When these aspects are taken together, even unweighted, narrow-ranged, human- and climate-stressed species should attain priority for listing. Based on this concept, for instance, the Class I *Teinopalpus aureus* would likely to be downgraded to Class II due to its wide distribution range in China [88], while *Losaria doubledayi* currently in the *EES List* would probably be listed as Class I due to its very restricted range only in Hainan Island [107].

One exhaustive but vital aspect of listing protected species is including synonymies (different names of a same species), especially the synonymies derived and widely used in China. A major problem of leaving synonymies unaddressed is biased judicial decisions involving protected species, if the valid name has been listed on the *SSP List* while its synonymies are found on the *EES List*. *Bhutanitis thaidina* and *B. mansfieldi* in West China are the most typical examples. Both species are currently listed as Class II species in the *SSP List* [86], but their synonymies, *B. yulongensis* (= *B. thaidina* populations in Yunnan), *B. nigrilima* (= *B. thaidina* populations in West Sichuan), and *B. pulchristriata* (= *B. mansfieldi* populations in West Sichuan) are all listed in the *EES List* [87,91]. In this case, illegal collection of these species in those areas could avoid penalties. Hence, ‘closing loopholes’ is necessary when formulating the list.

A feasible way to develop a reasonable list is to invite national-wide experts in taxonomy and conservation biology to evaluate the list every five years. The National Forestry and Grassland Administration and other related Ministries can devise an official form (preferably via online system) for the experts to complete. In this form, experts can note their opinions regarding certain species (e.g., adding/removing species, upgrading/downgrading ranks, etc.), as well as providing reasons and supporting literature. The decision-making expert panel in the Ministries can use the information to adopt final decisions about the lists.

#### 4.1.2. Identify High Priority Areas and Refugia

Two types of high priority areas exist: (1) areas with high butterfly species richness and (2) areas with species having higher conservation values (e.g., protected or endangered species). Understanding the spatial location and pattern of these areas can promote efficiency in conservation practices and better selection of survey sites that will produce the most useful information. Today such identification can be easily performed using multiple species distribution models (SDMs) [100,101,102,103,104,105] and GIS. With the development of base maps of species richness, researchers can conveniently overlap spatial information related to existing protected areas and human disturbances (e.g., land uses, wildfires, infrastructure construction, etc.) with the base maps to further identify conservation priorities. Another advantage of this research is that it also enables us to obtain a better understanding of potential refugia for certain species under climate change, which would be extremely useful when ex situ rescue or population reintroductions must be implemented [108,109,110]. Areas of high endemism should also be a future priority; the commonly applied parsimony analysis of endemicity affiliated with GIS could provide such vital information for conservation planning [111,112,113,114]. Recent research revealing the spatial character of pollinating butterflies in Yunnan could be a starting point [115].

Consideration might be given to revision of the *Wildlife Protection Law* to incorporate the concept of critical habitat. Wildlife authorities would designate the critical habitat for species appearing on the *SSP List* or the *EES List*. Critical habitat might be defined as physical or biological features essential for the conservation and survival of the listed species. Government agencies considering approval of projects in a critical habitat would be required to evaluate potential impacts of the proposal on the survival of the listed species and be prohibited from destroying or adversely modifying its critical habitat. The critical habitat concept would promote ecosystem-based management which is more inclusive than single-species management. It would also incorporate the concept of umbrella species that we discuss below in Section 4.1.4. The listed species could serve as an umbrella protecting other butterfly species in the area. The critical habitat protections could also be folded into the existing Environmental Impact Assessment process. The Critical Habitat management strategy appears in conservation legislation in Australia (Environment Protection and Biodiversity Conservation Act), the European Union (EU Habitats Directive), and the USA (Endangered Species Act).

#### 4.1.3. Assessing Genetic Diversity

Genetic diversity of a given species is vital for the formulation of good conservation strategies, especially when ex situ rescue or population translocations (including true translocations and reintroductions) are required in order to increase its fitness [109,116,117]. Population genetic research of endangered, endemic, and protected butterflies should be encouraged by forestry or environmental protection authorities on a regular basis or established as a component of standardized research packages in the conservation projects of those species. Quantifying population structure would benefit determining how management strategies, population histories (via monitoring data), and/or environments influence patterns of diversity [118,119,120,121,122,123,124,125,126]. Also, identifying levels and directions of gene flow and the number of private alleles in populations can provide critical information for a more effective conservation strategy in terms of increasing fitness [121,127,128,129,130,131,132]. With the advance of high throughput sequencing and non-lethal sampling technologies [133,134,135,136,137], carrying out regular monitoring of the temporal shifts in the genetic diversity of focal species is actually a cost-effective and butterfly-friendly method to accumulate large volume of data for future conservation strategies.

The other aspect of genetic diversity research is to determine the degree of population divergence, especially in species covering a wide geographical range with internal barriers. When genetic divergence among analysed populations reaches subspecies levels, conservation strategies must be formulated based on the population sizes and distribution ranges of the subspecies [107]. Priorities should be given to those with lower population sizes or narrower distribution ranges. A hidden usage of this evaluation is to detect cryptic species mixed in morphologically similar groups [107,138,139]. Misidentification of such species as subspecies would jeopardise future ex situ rescue involving translocations, as the environmental requirements for different species can vary significantly from those of different subspecies belonging to a same species. Moreover, including evolutionary perspectives in conservation planning could prevent potential domestication of the focal species [140].

#### 4.1.4. Using Umbrella Species to Boost Conservation

Conservation actions involve a series of practical procedures and decisions that must integrate costs; therefore, we cannot simply inflate the number of species on any protected list at any scale for the sole purpose of ‘better conservation’ [141]. However, the efficiency of biodiversity conservation can be boosted when umbrella species are properly identified and utilised [142]. To achieve this goal, biological research must be encouraged to obtain information about sympatric species sharing the same resources (e.g., vegetation types, habitats, or host plants). Species of regional iconic or faunistic representativity can serve as candidates for flagship (umbrella) species on future protected species lists. Table 1 shows some examples of umbrella effects via sharing larval food plants between the five species in the *SSP List* and other sympatric Papilionidae in China. More umbrella species of different butterfly families can also be identified adopting such criteria.

### 4.2. Wise Land Use Management

#### 4.2.1. Butterfly-Friendly Agriculture

**Figure 1:**
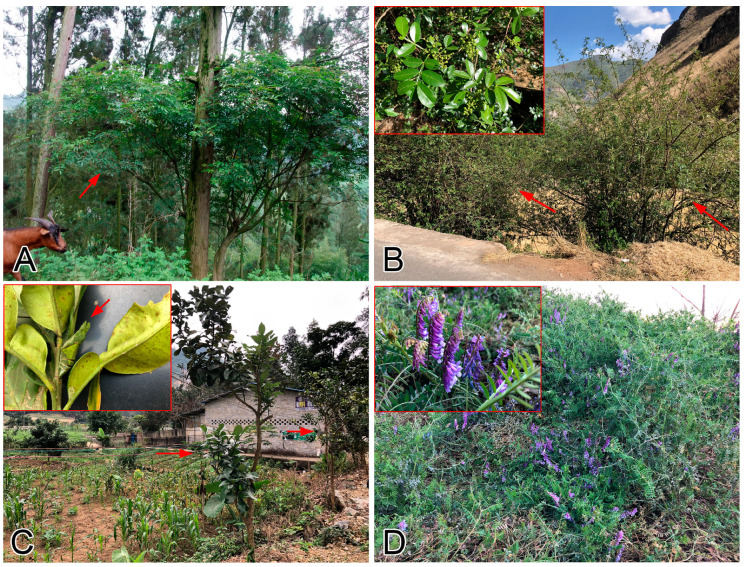
Examples of butterfly attracting plants in Chinese agroecosystems, cited from Zhang, Wang, Yu, Xing, Xu, Duan, Zhu, Zhang, Li and Hu [115]. (**A**): A *Zanthoxylum* bush (red arrow); (**B**): *Zanthoxylum* bushes as fence (red arrows), with fruiting branch in the red box; (**C**): *Citrus maxima* (pomelo) along the edge of field (red arrows), with a pupa of *Papilio memnon* attached to a branch in the red box (red arrow); (**D**): *Vicia* sp. during the seasonal rotation, with close up of flowers in the red box.

Monoculture, introduction of alien plant species, and the inevitable application of PPPs are three major causal factors for diversity declines in agroecosystems, and many essential pollinators (including butterflies) often become the collateral damage in such ecosystems [2,143,144,145,146,147]. It has been demonstrated that changes in land use management (e.g., increasing landscape heterogeneity, control of alien plant species, introduction of butterfly attracting plants, maintenance of unmanaged hedges, decreased use of PPPs/organic farming, etc.) can solve the dilemma and increase the diversity, abundance, and ecological service functions of butterflies in agroecosystems worldwide [115,148,149,150,151,152,153,154,155,156]. In fact, these measures are not completely new in Chinese agricultural practices, although they have yet to be formally recognized as best management practices. In Yunnan and Sichuan provinces, farmers traditionally grow *Zanthoxylum* bushes or pomelo trees as fences around fields (Figure 1A), which serve as the larval food plants for at least five species of *Papilio* butterflies [52]. The fruits of these *Zanthoxylum* bushes are a widely used spice called *huajiao* (prickly ash), further adding farmers’ income. Fruits of pomelo also bring in economic benefits. Herbaceous plants such as *Vicia* are more widely planted in vacant fields during seasonal rotation (Figure 1B), providing larval food plants for the widespread *Colias poliographus*, *C. fieldii*, and *Lampides boeticus* across China [52], as well as a nectar source for various sympatric butterflies. Additionally, the plants themselves are capable of nitrogen fixation and can later be ploughed into the soil to improve soil quality. The following list contains a few examples of candidate butterfly attracting plants that can increase pollinator diversity and benefit farmers in agroecosystems (Table 2). Maintaining unmanaged hedges between fields is also a positive measure, as most annual *Poaceae* plants (native grasses) are able to support a good number of Hesperiidae butterflies [52], while the flowering plants (e.g., *Bidens* spp.) can be excellent nectar sources to attract pollinators. Introduction of butterfly attracting plants into agroecosystems offers additional benefits of indirectly increasing landscape heterogeneity, overall crop-pollinator interactions, and the number of natural enemies to agricultural pests [157,158].

#### 4.2.2. Butterfly-Friendly Urban Green Spaces

**Figure 2:**
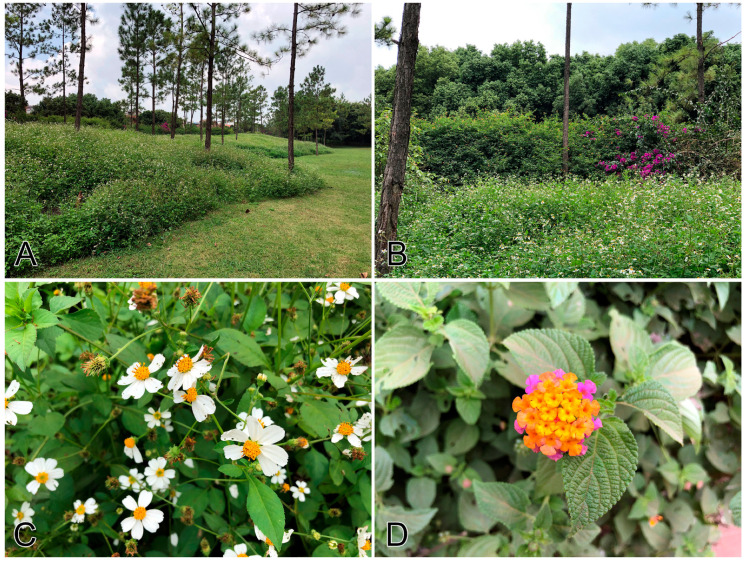
Unmanaged green space in the Huquan Park of Mile (a very young city), Yunnan Province, Southwest China. (**A**): boundary between managed lawn and unmanaged green space; (**B**): plant community inside the unmanaged green space; (**C**): dominant nectar source *Bidens* sp.; (**D**): nectar source *Lantana camara* along the road.

Wise land use and floristic planning in green space can serve as refugia for butterflies inside urban areas. Multiple studies have revealed that unmanaged hedges in urban parks; uncommitted weedy patches along river banks, roadsides and medium strips; a diversity of native butterfly attracting plants—both larval host and nectar plants; and landscape heterogeneity are more favorable for butterfly diversity than carefully managed green spaces, alien or monocultural plants, and monotonous landscapes [2,54,159,160,161,162,163,164]. Our 15-year observations in the downtown areas in Kunming also showed similar patterns; butterfly diversity in less managed mosaic habitats is significantly higher than the carefully managed monotonous ones (Table 3). Under the guidance of the ‘ecological civilisation’ concept, recent advances in urban planning have begun to build ‘ecological parks’ in new cities with a good proportion of native/original vegetation maintained as unmanaged green space (Figure 2). Older cities, like Beijing, have begun to re-establish insect-friendly green spaces with insect-attracting plants, artificial nesting sites (rocks and plant litters), and reduction of PPP application [165].

#### 4.2.3. Increasing Diversity in Reforestation

Over the past five decades, China has invested significant efforts in reforestation to compensate for the historical loss of forest resources [166,167]. Increasing forest cover versus maintaining biodiversity in reforested areas has been a debated topic in forest policy [168,169,170]. Monoculture of selected tree species is often a typical image in reforestation, especially in projects like sand-fixation and slope conservation in China [60]. However, this reality should not be interpreted as evidence that China’s reforestation policies ignored the importance of diversity. Even back in the 1960s, Chinese forestry administrations had already begun to encourage use of a diverse range of native plants in reforestation in order to maintain sufficient biodiversity in artificially created and maintained forests; unfortunately, this ecological ideal has faced many constraints (e.g., budgetary shortages, commercial interest in timber harvests, lack of manpower for irrigation, etc.), often resulting in forest monocultures because only a limited number of ‘suitable’ trees can thrive after sowing or planting [40,59,60]. With the evolving appreciation of biodiversity and the advance of reforestation technologies, the use of a variety of native tree species in reforestation is widely adopted in China today [169,171,172,173].

Despite the debate, many studies illustrate the ecological benefits of reforestation. Reforestation not only increases forest cover, but also provides a better shelter for understory fauna; research has demonstrated higher species richness and abundance of ants, beetles, birds, and small mammals in reforested areas [174,175,176,177,178,179,180,181]. Similar studies in the megacity of Beijing also illustrate that the diversity birds and beetles in reforested areas is higher [182,183,184]. Moreover, research using these organisms as ecological indicators suggests that biodiversity and ecological function performance in reforested areas using a variety of native plants are generally higher than those efforts using exotic or selected monocultures [185,186,187,188,189]. Although analyses of butterfly diversity in reforested areas is scarce, it is logical to speculate that reforestation with various native plants could also enhance butterfly diversity. Future studies on butterflies’ responses to different plantation strategies will provide new insight into how to further biodiversity conservation during reforestation.

#### 4.2.4. Protection of Traditional Forests

Traditional ecological knowledge (TEK) often offers great environmental benefits and should be respected, studied, and implemented where applicable in new situations. For example, in southern China, small forest patches with heterogeneous habitats often exist adjacent to villages and are protected by the community residents [190,191]. These culturally protected forests are manifestations of traditional Chinese spatial concepts of beauty and are referred to as village *fengshui* forests. As heterogeneous habitat spaces, they likely harbour greater insect diversity than surrounding areas and certainly promote regional biodiversity [192,193]. Further research will demonstrate the relationship between these culturally protected forests and butterfly diversity.

### 4.3. Pros and Cons of Butterfly Ranching and Collecting

#### 4.3.1. Ranching over Collecting

**Figure 3:**
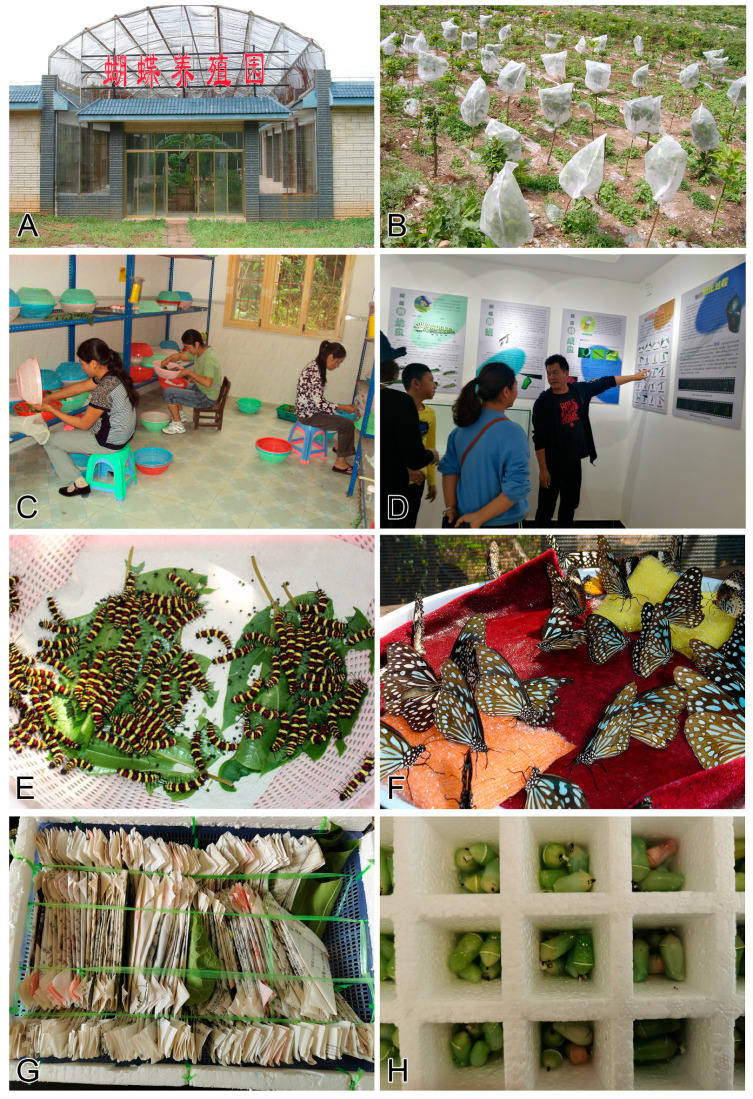
Butterfly ranches in China. (**A**): Ranch greenhouse in Yunnan; (**B**): Ranch garden in Hainan; (**C**): Local women employed in the Yunnan butterfly ranch; (**D**): Ranchers in Yunnan explain butterfly life cycles to the public; (**E**): Larvae of reared *Cethosia cyane* (Heliconiinae); (**F**): Adults of reared *Tirumala limniace* (Danainae); (**G**): Packed live adults ready for shipping; (**H**): Packed live pupae (Danainae) ready for shipping.

When non-scientific butterfly consumption (ornamental artwork and ceremonial release) remains legal, it is illogical to completely ban butterfly collecting because butterflies provide sustainable household income in many rural areas, especially in the tropics. However, the stress on wild butterflies could be effectively alleviated by encouraging butterfly ranching or farming, because non-scientific consumption only requires a limited range of species which can easily cultivated by mass production in greenhouses [194]. In recent years, four certificated butterfly farms have been established in various places in Yunnan, Guangxi, and Hainan Provinces (Figure 3) (X.M. Chen & J. Yao, pers. comm.). The traditional ‘products’ of these farms include several species of large, beautiful Papilionidae and many long-lived Nymphalidae (especially Danainae) species, mainly sold as pupae in cities for ceremonial release or tourist butterfly gardens. With the growing demands for butterfly species, producers work with lepidopterists to explore methods for the cultivation of more species gathered from nature. In order to maintain a sustainable business, butterfly ranchers must conserve the local butterfly resource and environment [195,196,197]. Butterfly farms may also supplement local residents’ incomes, and thus, avoid environmentally harmful activities, e.g., deforestation [198]. If supported by government policies, butterfly ranching may gradually come to substitute collection of some species. Conservation benefits can also ensue with requirements that each farm release a certain proportion of its production (native species only). Today the butterfly ranchers voluntarily release about 10% of their production.

Successful examples of butterfly ranching and farming that integrate community development and socio-economic benefits exist throughout the world. The classic case is Papua New Guinea (PNG), a country with extremely high butterfly diversity. In the 1960s the PNG government granted protected status to seven butterfly species and a fostered small-scale butterfly ranching industry and trade opportunities based on several birdwing species. Ranching provides income to local communities, promotes habitat conservation through protection of host and larval plants, release of a high percentage of adults, as well as avoided deforestation. New [2] stressed the importance of centralized government coordination of this activity to cap the number of farms, ensure compliance with regulations, and avoid illegal capture and trade of protected species.

**Figure 4:**
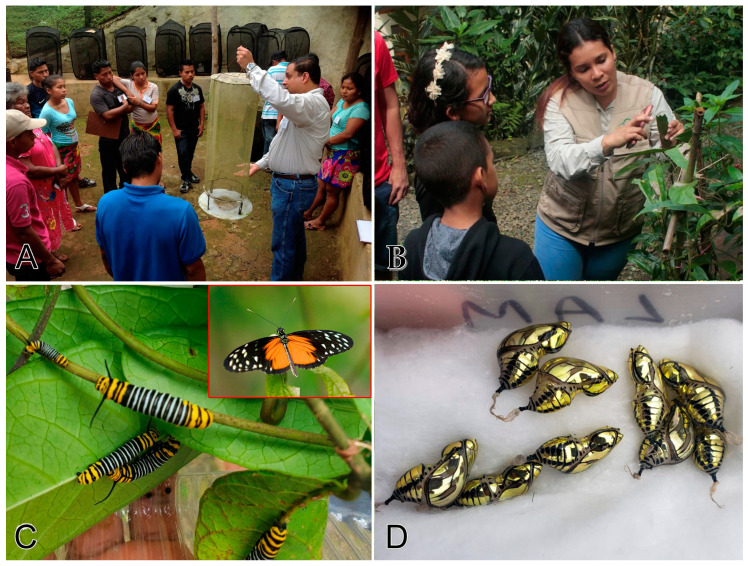
Scenes of Mariposario Cerro La Vieja (Butterfly Farm) in Panama. (**A**): Samuel Valdés, owner of the Mariposario Cerro La Vieja (Butterfly Farm), Panama, conducting a training in butterfly ranching methods; (**B**): Marelys Torres, co-owner of the Mariposario Cerro La Vieja, explaining butterfly life cycles to local students; (**C**): Larvae of *Tithorea tarricina* (Ithominae) feeding on host plant *Prestonia portobellensis* (Apoacynaceae), with an perching adult in the red box; (**D**): Pupae of *T. tarricina* (Ithominae) packed for shipment to Panama City.

Recent noteworthy experiences with butterfly ranching in other developing countries (Figure 4), e.g., Costa Rica [199,200], Ecuador [201], El Salvador [202], Tanzania [203], all demonstrating the synergies between business and job opportunities for local communities (particularly women), community development initiatives, environmental benefits (avoided deforestation, establishment of private nature reserves, protection of host and nectar plants), sale and export of pupae, and opportunities for public environmental education, outreach, and ecotourism. Costa Rica, for example, boasts an extended network of about 400 butterfly farms distributed in diverse habitats throughout the small Central American nation [199].

An international network of butterfly ranchers and exhibitors has existed for over a decade. The International Association of Butterfly Exhibitors and Suppliers (IABES) [204] promotes sustainable butterfly exhibitions and suppliers, while supporting conservation efforts and public education. Connecting sustainable suppliers and exhibitors, IABES also advises exhibitors with their educational plans and offers advice to butterfly ranchers about sustainable practices. IABES also serves as a communication hub between its members, researchers, the media, and regulatory bodies.

#### 4.3.2. Developing and Enforcing Regulations for Collecting

Butterfly collection may be a necessity for scientific researchers. Purchasing butterfly specimens from dealers may become cost-effective when demands cannot be met by butterfly ranching or when visiting certain localities is expensive or impossible. Specimens from a certain locality or variety are usually very valuable—particularly protected or endangered species (A.M. Cotton & Y.F. Hsu, pers. comm.). The possibility of high profits motivates dealers and local collectors to seek those particular specimens for sale [93]. One possible way to protect butterflies from over-collection is the creation of more sophisticated regulations to control collection, based on the framework of the current *Wildlife Protection Law* [48]. Under such regulations, only authorised persons can collect an approved quantity of a certain butterfly species during certain times of the year after payment of a conservation fee. Additionally, specimens collected under these permits can be freely traded or exchanged. Meanwhile, authorities should enforce the complete prohibition of illegal collection, and persons collecting or possessing illegally obtained specimens should face strict judicial penalties. Regulations should be revised to discourage collection of wild butterflies using clearer legal definitions while providing the necessary space for scientific research, as well as for professional collectors who collaborate with researchers.

### 4.4. Increasing Public Involvement

#### 4.4.1. Adoption of Citizen Science

Citizen science involves integrating the public into research and data collection. Numerous examples of citizen science programs illustrate numerous advantages and positive outcomes: (1) data can be collected over large spatial and temporal scales, permitting measurements of global land use and climate changes and trend analyses, (2) research and data collection costs may be significantly reduced and the data pool greatly expanded, (3) participants may develop a keener connection to the environment, understanding of scientific methodologies and knowledge, and familiarity with conservation challenges, and (4) in some cases, participation in a project may encourage citizen scientists to take further actions on conservation issues [205,206,207,208,209]. To be successful, citizen science programs require simple standardized observation protocols, measures to assure quality control, expanded data handling techniques, and regular feedback to observers.

Butterflies are excellent candidates for citizen science programs. They are attractive and charismatic, easily observed during the daytime, and good indicators of environmental change because of their short lifespan and mobility. Many citizen science monitoring programs use the internet and location-aware smartphone applications to record butterfly observations (time, place, number, life stage, and photograph) and verify species. Numerous examples exist of butterfly monitoring programs involving citizen scientists; European countries, North America, Malaysia, Singapore, India, and Japan have widely adopted citizen scientist programs under well-established guidelines for monitoring butterfly diversity [210,211,212,213,214,215,216,217,218,219].

During the past 50 years, the UK nation-wide observation network has accumulated massive butterfly occurrence data and created fine-grid diversity distribution maps that scientists and conservationists can utilize to periodically assess diversity changes. Since 1976 the UK Butterfly Monitoring Scheme [210] has compiled data on 71 species of butterflies and been able to assess status and trends in populations for conservation and research purposes. Additionally, the UK’s Big Butterfly Count is the world’s biggest survey of butterflies; some 113,500 citizen scientists took part in 2019 and submitted 116,009 counts of butterflies and day-flying moths [220].

The outstanding North American experience in butterfly citizen science involves the monarch (*Danaus plexippus*) and tracking its breeding and seasonal migration between Canada, the USA, and Mexico. Ries and Oberhauser [221] report that of the 503 research publications focused on monarchs from 1940–2014, 17% used citizen science data. Today numerous monarch citizen science programs are coordinated under the Monarch Joint Venture [222]. An additional program broader in species scope is “eButterfly” [219]. This program uses a standardized checklist to document the presence or absence of North American butterfly species. Species distribution data is open access and participants can enjoy incentives of access to species information and real-time distribution maps, discussion of sightings with other participants, and sharing of butterfly photos.

A good example of citizen participation in butterfly conservation in China is from Taiwan, local communities joined the butterfly monitoring programme voluntarily while working under unified guidelines [223,224]. Such collaboration between academic and citizen scientists should also be encouraged in mainland China, where the number of private butterfly researchers is growing every year. Today, private butterfly enthusiasts in Beijing, Jiangsu, Guangdong, and Yunnan provinces have already participated in the China BON-Butterflies programme. Policies to support increased citizen scientist participation in the butterfly observation network should be created to make the China BON-Butterflies programme a more powerful platform for butterfly conservation. These policies may focus on flexible funding systems and other motivational mechanisms (e.g., access to data and information via websites and newsletters, certificates that recognize participation, ability to participate in research design, etc.) to reward and incentivize citizen scientists.

#### 4.4.2. Elevation of Public Awareness

Public awareness and outreach are the base upon which butterfly conservation efforts in China depend. Apart from conventional publicity measures used in the protection of other flagship species, additional methods must be explored and implemented to bring butterflies to the attention of the public based on their intrinsic values and aesthetics, cultural values and place in tradition, the ecological services they provide, potential economic benefits, and the threats they face. Formal education has a role in learning about butterflies via videos and instructional materials, student campaigns, classroom museums, and social networking webpages; additional materials (e.g., videos, children’s butterfly observation books, puzzles, games) may play roles in informal education. Professional and citizen scientists must also collaborate to translate scientific findings into vehicles more easily understandable and interesting for the general public, such as pocket guide books for butterflies of a region, presentations, or media articles [225]. Campaigns should also target farmers via visits by agriculture extension agents, brochures, and media programs on best management practices for gardening and landscaping. Butterfly houses and gardens by themselves or as components of zoos and botanical gardens can raise awareness, as well as promoting ecotourism and butterfly ranching. Smartphone applications and internet guides (e.g., iNaturalist—global, iButterflies—India, Big Butterfly Count—UK, eButterfly—North America) can also assist butterfly field identification.

## 5. Conclusions

Butterflies offer numerous services—ecosystem, economic, and cultural—and, in addition, are important indicators or ecosystem health, as well as changes in land use and climatic conditions. The People’s Republic of China spans a wide variety of terrestrial habitats, ranging from high altitude mountains to temperate deserts and tropical rainforests. As a result, the nation’s butterfly diversity is high, and many regions count narrow-range endemic species.

In recent decades, China has experienced tremendous economic growth that has caused significant pressures on the natural environment. Global climate change is an additional driver affecting species distributions and biodiversity. China’s environmental protection and conservation policies have evolved considerably in recent decades. Notably, national policies emphasizing sustainable development and environmental protection—’ecological civilisation’—have taken a central stage in national decision making. The China National Biodiversity Conservation Strategy and Action Plan (2011–2030) provides the framework for conservation actions at all levels of government and, as well, meets China’s commitment to implementation of the Convention on Biological Diversity. Within this framework the Ministry of Ecology and Environment is currently implementing the China-BON Butterflies programme to assess these species’ biodiversity.

Our essay examines some of the challenges facing butterfly conservation in China today. Based on our evaluations, we recommend numerous practical initiatives that China could adopt to improve butterfly conservation and advance the national biodiversity conservation goals. The recommendations may be implemented by combinations of government authorities, scientists, and local communities. We categorize our recommendations into those that focus on improvement of conservation science and management (protected species listings, identification of high priority areas for protection, use of umbrella butterfly species, more explicit regulations for butterfly collection), better land management (agricultural areas, urban spaces, forests), promotion of butterfly ranching and farming, and encouragement of citizen participation in conservation efforts (citizen science and public outreach and education).

In Table 4 we list the ten Priority Areas of the China National Biodiversity Conservation Strategy and Action Plan (2011–2030) and our recommendations for improving butterfly conservation. We indicate the principal Priority Areas that our recommendations address. China’s conservation scientists and managers have already taken major steps toward protecting the nation’s butterfly species. Implementation of our recommendations will help advance the conservation of this important resource.

## Figures and Tables

**Table 1:** Unprotected Papilionidae species sheltered under umbrella species (in the protected *SSP List*) via sharing larval food plants.

Protected Species	Larval Food Plants	Sheltered Species
*Teinopalpus aureus*	*Magnolia* spp., *Michelia* spp. (magnolia)	*Teinopalpus imperialis*, *Graphium agamemnon*, *G. doson*, *G. chironides*
*Bhutanitis thaidina* and *B. mansfieldi*	*Aristolochia* spp. (birthwort, pipevine, Dutchman’s pipe)	*Byasa plutonius*, *B. nevilli*, *B. latreillei*, *B. polla*, *B. hedistus*, *B. polyeuctes*, *B. daemonius*, *B. rhadinus*, *B. confusa*, *B. impediens*, *Pachliopta aristolochiae*, *Troides aeacus*, *Bhutanitis lidderdalii*
*Luehdorfia chinensis*	*Asarum* spp. (wild ginger)	*Luehdorfia longicaudata*
*Parnassius apollo*	*Rhodiola* spp. (golden root, rose root)	*Parnassius nomion*, *P. epaphus*, *P. mercurius*, *P. actius*, *P. tianschanicus*, *P. apollonius*

**Table 2:** Examples of butterfly attracting plants and their known economic benefits.

Plants	Related Butterflies	Function	Economic Benefits
*Zanthoxylum* spp. (prickly ash or huajiao)	*Papilio xuthus*, *P. bianor*, *P. maackii*, *P. helenus* (feeding)	food plants	fruits as spice
*Citrus maxima* (pomelo)	*Papilio memnon*, *P. protenor*, *P. polytes*, *P. demoleus* (feeding)	food plants	fruits for market or family consumption
*Vicia* spp. (vetches)	*Colias poliographus*, *C. fieldii*, *Lampides boeticus* (feeding); *Papilio* spp., *Byasa* spp., *Pieris* spp., *Pontia* spp., *Gonepteryx* spp., *Tirumala* spp., *Parantica* spp., *Vanessa* spp., *Heliophorus* spp., Hesperiidae (flower visiting)	food plants, nectar sources	nitrogen fixation, whole plant as green manure improving soil quality
*Brassica rapa* (field mustard)	*Pieris rapa*, *P. canidia* (feeding) *Papilio machaon*, *Colias* spp., *Pontia daplidice*, *Heliophorus* spp., *Ahlbergia* spp. (flower visiting)	food plants, nectar sources	flowers for bee keeping and honey production, fruits for the oil industry
*Tagetes erecta* (marigold)	*Papilio* spp., *Pieris* spp., *Pontia daplidice*, *Colias* spp., *Gonepteryx* spp., *Danaus* spp., *Tirumala* spp., *Parantica* spp., *Argynnis* spp., *Vanessa* spp., Hesperiidae (flower visiting)	nectar source	flower as material for the carotene industry

**Table 3:** Inhabiting butterfly fauna observed in four types of urban habitats in Kunming downtown area.

Habitat	Habitat Type	Common Butterfly Species
Kunming Zoo	Mostly unmanaged green space with sparse animal keeping areas	*Graphium sarpedon*, *G. cloanthus*, *Papilio bianor*, *P. xuthus*, *P. polytes*, *Ixias pyrene*, *Cepora nerissa*, *Hebomoia glaucippe*, *Pieris rapae*, *P. melete*, *Prioneris thestylis*, *Delias belladonna*, *Appias albina*, *Catopsilia pomona*, *Eurema laeta*, *E. hecabe*, *Gonepteryx chinensis*, *G. amintha*, *Danaus chrysippus*, *D. genutia*, *Parantica sita*, *P. swinhoei*, *Tirumala septentrionis*, *Euploea mulciber*, *Vanessa cadui*, *V. indica*, *Hypolimnas bolina*, *Hestina persimilis*, *Apatura ilia*, *Polyura dolon*, *Tongeia ion*, *Celastrina oreas*, *Lampides boeticus*, *Jamides bochus*
Yunnan University	Carefully managed campus with unmanaged green spaces along hill slopes	*Papilio bianor*, *P. xuthus*, *Ixias pyrene*, *Cepora nerissa*, *Appias albina*, *Catopsilia pomona*, *Eurema laeta*, *Parantica sita*, *Vanessa cadui*, *Hestina persimilis*, *Tongeia ion*
Kunming street type 1	managed street with various camphor trees	*Graphium sarpedon*, *G. cloanthus*
Kunming street type 2	managed street with jacaranda trees	no resident butterfly species

**Table 4:** Overlaps between our Recommendations and the Priority Areas of the China National Biodiversity Conservation Strategy and Action Plan (2011–2030).

Priority Areas	Recommendations	Priority Areas Targeted by The Recommendation
1-Improve the policy and legal system of biodiversity conservation and sustainable use	Revise the Protected Species Lists	1, 3, 4
2-Incorporate biodiversity conservation into sectoral and regional planning and promote sustainable use	Identify High Priority Areas and Refugia	2, 3, 4, 8, 9
3-Identify, evaluate, and monitor biodiversity	Use Umbrella Species to Boost Conservation	4
4-Strengthen in situ biodiversity conservation	Encourage Butterfly-friendly Agricultural Methods	2, 4, 6, 10
5-Carry out ex-situ conservation based on science	Promote Butterfly-friendly Urban Spaces	2, 4, 10
6-Promote rational use and benefit sharing of biological genetic resources and associated traditional knowledge	Increase Diversity in Reforestation Programmes	2, 4
7-Strengthen biosafety management of invasive alien species and genetically modified organisms	Protect Traditional Forests	2, 4, 6, 10
8-Improve capacities to cope with climate change	Promote Appropriate Butterfly Ranching and Farming	4, 5, 6, 10
9-Strengthen scientific research and human resources development in the field of biodiversity	Develop and Enforce Regulations for Butterfly Collection	1, 6, 9, 10
10-Establish public participatory mechanisms and partnerships for biodiversity conservation	Adopt Citizen Science Campaigns	3, 4, 7, 9, 10
	Promote Public Awareness and Educational Opportunities	10
	Revise the *Wildlife Protection Law* to incorporate the Critical Habitat concept	1, 4
